# Editorial: Molecular, structural and electrophysiological remodeling in atrial fibrillation

**DOI:** 10.3389/fphys.2023.1303328

**Published:** 2023-10-12

**Authors:** Diego Franco, Leif Hove-Madsen, Houria Daimi

**Affiliations:** ^1^ Cardiovascular Development Group, Department of Experimental Biology, University of Jaén, Jaén, Spain; ^2^ Fundación Medina, Granada, Spain; ^3^ Cardiac Rhythm and Contraction Group, Department of Experimental Pathology, Biomedical Research Institute of Barcelona (IIBB-CSIC), Barcelona, Spain; ^4^ Biomedical Research Institute Sant Pau, (IIB, Sant Pau), Barcelona, Spain; ^5^ CIBER Enfermedades Cardiovasculares (CIBERCV), Instituto de Salud Carlos III, Madrid, Spain; ^6^ Laboratory of Human Genome and Multifactorial Diseases (LR12ES07), Faculty of Pharmacy, University of Monastir, Monastir, Tunisia; ^7^ Department of Biology, Faculty of Sciences, University of Gabès, Gabès, Tunisia

**Keywords:** atrial fibrillation, cardiomyocyte remodelling, fibrosis, inflammation, arrhythmia, intracellular Ca2+ handling, ion channels, signalling cascades

Atrial fibrillation (AF) is the most common clinically relevant arrhythmia. Growing evidence indicates that AF is a progressive disease of which the occurrence, maintenance and progression results from molecular, electrophysiological and structural remodeling. Altogether they will lead to substantial complications that significantly contribute to population morbidity and mortality. Although important progress has been made, the mechanisms involved in the changes that occur in the atria of AF patients are not yet fully understood. Thus, further studies focusing on delineating these mechanisms in humans, as well in AF animal models are needed in order to develop and exploit novel therapeutic avenues and to prevent the progression of the arrhythmic diseases. This Research Topic brings together four distinct papers introducing new AF mice models and shedding light on some molecular mechanisms underlying AF.

In this Research Topic, Trieu et al. aimed to generate a new genetically unbiased AF murine model by selectively sensitizing atrial muscle to diphtheria toxin (DT), a molecule that induces cell damage. For this goal, they have crossed the atrial-specific Sarcolipin (Sln)-Cre knock-in mouse (*Sln* ^*+/Cre*^) with the iDTR mouse (*R26* ^*+/DTR*^). The new model was characterized by paroxysmal and persistent AF as well as atrial myocardial fibrosis. The presented data demonstrated that more than half of the DT treated Sln^+/Cre^; R26^+/DTR^ mice developed AF within 2 months of treatment and that the severity of atrial damage in this model is dose and time dependent. DT injected Sln^+/Cre^; R26^+/DTR^ with confirmed AF reverted to the sinus rhythm after being treated with amiodarone suggesting that this new animal model closely mimics clinical features of AF and that it presents a new tool to understand AF mechanisms and develop new therapeutics.

Based on their previous study in which they correlated Ang II mediated hypertension with increased AF susceptibility and the related structural and electrical remodelling in Ang II infused mice (Jansen et al., 2018), Jansen et al. investigated the regional and temporal progression of these changes and the resulting AF predisposition. Accordingly, mice were chronically infused with Ang II for 3, 10 and 21 days and the effects of Ang II on atrial function were explored electrophysiologically and histologically. Ang II infused mice displayed progressive changes in atrial electrophysiological parameters (i.e., P wave duration, AERP) as early as after 3 days of infusion and AF manifestation after 10 days of Ang II suggesting that changes in atrial electrophysiology must progress to a threshold before an overt increase in AF susceptibility becomes clear. Left, but not right atrial myocyte hypertrophy in addition to progressive right and left atrial fibrosis was also observed after 3 days of Ang II infusion. These data provide an interesting insight into the patterns of atrial remodeling that lead to increased AF susceptibility.

Guillot et al. investigated the involvement of EPAC proteins, cAMP binding proteins, in the genesis of AF. Accordingly, they employed mouse models with deletion of EPAC1 or EPAC2 isoforms and pharmacologically treated with 8-CPT-AM to activate the EPAC pathway or with AM-001 and ESI-05 to selectively inhibit EPAC1 and EPAC2, respectively. Treated mice were electrophysiologically characterized and tested for AF inducibility and incidence. The results indicate that EPAC1 and EPAC2 activation increases AF susceptibility by prolonging the action potential duration (APD) and decreasing the conduction velocity (CV) in atria. These data confirm the paradoxical involvement of APD increase in the pathophysiology of AF where both proarrhythmic and antiarrhythmic effects are described. While APD prolongation by EPAC has been attributed to a decrease in steady state K (+) current in ventricular myocytes (Brette et al., 2013), Guillot et al. failed to explain this prolongation in their AF model. Nonetheless, they suggested that the decreased conductance is likely due to connexin remodeling. Interestingly, only the EPAC1 isoform and its inhibitor, AM-001, are involved in AF genesis or prevention, respectively, introducing thus novel and promising therapeutic target and tools in AF.

Finally, Zhao et al. brought new insights into the role of RNA internal methylation modification at the N6 position of adenosine (m^6^A) in AF susceptibility. In this study, the authors screened publically available datasets of AF and control atrial samples expression microarrays in search of any correlation between AF incidence and expression of m^6^A regulatory genes. Their data revealed that among the extracted m^6^A regulatory genes, three (IGFBP2, YTHDF1, and IGFBP3) were upregulated and two (ZC3H13 and HNRNPA2B1) were downregulated in the AF samples, compared to controls, highlighting their potential biological significance in AF onset and progression. In addition, m^6^A regulatory genes displayed significant positive (i.e., HNRNPA2B1 vs. METTL3 and YTHDF2 vs. FTO) or negative (YTHDC2 vs. RBM15B) correlation between each other. Particularly, AF patients with a high level of ALKBH5 gene showed low expression levels of HNRNPA2B1 and METTL3 genes. Furthermore, samples with higher IGFBP3 expression presented an increased immune cell infiltration implying that m^6^A may be involved in AF development by regulating immune infiltration.

Taken together, the studies published in this Research Topic significantly increased our understanding about the mechanisms behind molecular, structural and electrophysiological remodelling in AF either by introducing new murine models to study AF or establishing predictive models that may all guide the exploration of molecular mechanisms contributing to the development of AF. Particularly, the described AF mimicking experimental models will serve not only in evaluating the efficacy of AF drugs/moderators but also the safety of the applied doses before moving into large scale clinical trials.

## References

[B1] Brette F. Blandin E. Simard C. Guinamard R. Sallé L. (2013). Epac activator critically regulates action potential duration by decreasing potassium current in rat adult ventricle. J. Mol. Cell Cardiol. 57, 96–105. 10.1016/j.yjmcc.2013.01.012 23376036

[B2] Jansen H. J. Mackasey M. Moghtadaei M. Belke D. D. Egom E. E. Tuomi J. M. (2018). Distinct patterns of atrial electrical and structural remodeling in angiotensin II mediated atrial fibrillation. J. Mol. Cell Cardiol. 124, 12–25. 10.1016/j.yjmcc.2018.09.011 30273558

